# Functional Contributions of the Arcuate Fasciculus to Language Processing

**DOI:** 10.3389/fnhum.2021.672665

**Published:** 2021-06-25

**Authors:** Maria V. Ivanova, Allison Zhong, And Turken, Juliana V. Baldo, Nina F. Dronkers

**Affiliations:** ^1^Aphasia Recovery Lab, Department of Psychology, University of California, Berkeley, Berkeley, CA, United States; ^2^Center for Language, Imaging, Mind & Brain, VA Northern California Health Care System, Martinez, CA, United States; ^3^School of Medicine, New York Medical College, Valhalla, NY, United States; ^4^Department of Neurology, University of California, Davis, Davis, CA, United States

**Keywords:** aphasia, stroke, language, diffusion MRI, tractography, arcuate fasciculus

## Abstract

Current evidence strongly suggests that the arcuate fasciculus (AF) is critical for language, from spontaneous speech and word retrieval to repetition and comprehension abilities. However, to further pinpoint its unique and differential role in language, its anatomy needs to be explored in greater detail and its contribution to language processing beyond that of known cortical language areas must be established. We address this in a comprehensive evaluation of the specific functional role of the AF in a well-characterized cohort of individuals with chronic aphasia (*n* = 33) following left hemisphere stroke. To evaluate macro- and microstructural integrity of the AF, tractography based on the constrained spherical deconvolution model was performed. The AF in the left and right hemispheres were then manually reconstructed using a modified 3-segment model (Catani et al., 2005), and a modified 2-segment model (Glasser and Rilling, 2008). The normalized volume and a measure of microstructural integrity of the long and the posterior segments of the AF were significantly correlated with language indices while controlling for gender and lesion volume. Specific contributions of AF segments to language while accounting for the role of specific cortical language areas – inferior frontal, inferior parietal, and posterior temporal – were tested using multiple regression analyses. Involvement of the following tract segments in the left hemisphere in language processing beyond the contribution of cortical areas was demonstrated: the long segment of the AF contributed to naming abilities; anterior segment – to fluency and naming; the posterior segment – to comprehension. The results highlight the important contributions of the AF fiber pathways to language impairments beyond that of known cortical language areas. At the same time, no clear role of the right hemisphere AF tracts in language processing could be ascertained. In sum, our findings lend support to the broader role of the left AF in language processing, with particular emphasis on comprehension and naming, and point to the posterior segment of this tract as being most crucial for supporting residual language abilities.

## Introduction

The arcuate fasciculus (AF) is a white matter bundle that connects the frontal and temporal lobes within each cerebral hemisphere by passing dorsally beneath the parietal lobe. The AF was already included in the first classic model of language processing proposed by Wernicke at the end of the 19th century (Wernicke, 1874; Lichtheim, 1885; Dronkers et al., 2017), and today it is widely acknowledged as the most crucial tract for language processing (Geschwind, 1970; Dronkers et al., 2000, 2007; Bates et al., 2003; Catani et al., 2005; Bernal and Ardila, 2009). Overall, studies of the critical role of fiber pathways in supporting language processing have been recently revived with the development of neuroimaging techniques that allow for *in vivo* visualization of white matter connections. Subsequently, connections between cortical areas have become as critical as the areas themselves in modern models of language processing (Turken and Dronkers, 2011; Dick and Tremblay, 2012; Friederici and Gierhan, 2013; Duffau et al., 2014; Bajada et al., 2015; Corbetta et al., 2015; Ivanova et al., 2016; Kiran and Thompson, 2019).

Though the AF was originally purported to support repetition of verbal information (Wernicke, 1874), contemporary dorsal-ventral models of language processing proclaim that the dorsal stream (anatomically supported by the AF complex) is critical for more general sensory-motor mapping of sound to articulation (Saur et al., 2008; Bornkessel-Schlesewsky et al., 2015). The dorsal stream has been proposed to provide an interface between the acoustic speech sensory system and the motor-articulatory system at the level of individual speech segments (basic articulatory phonetic skills) and sequences of segments (acquisition of new vocabulary and online guidance of speech sequences). Subsequently, beyond being involved in repetition (Hickok and Poeppel, 2007; Roelofs, 2014), it also supports production of complex syntax when successive processing is crucial (Friederici and Gierhan, 2013; Bornkessel-Schlesewsky et al., 2015). In contrast, the ventral stream (anatomically supported by long associative temporal lobe fibers) is hypothesized to support the mapping of sound to meaning (Hickok and Poeppel, 2007; Friederici and Gierhan, 2013; Bornkessel-Schlesewsky et al., 2015). However, it may be an oversimplification to think that the role of the AF is limited exclusively to repetition and production of complex syntax.

Numerous recent investigations in clinical populations underscore a more multifaceted role of the AF in language processing. Ivanova et al. (2016) demonstrated in a large cohort of individuals with post-stroke aphasia that different segments of the AF are related to different components of language processing, with the posterior temporal part of the tract associated with both comprehension and production at the word and sentence level. Fridriksson et al. (2018), in another large study of chronic stroke survivors, showed that components of language comprehension and production cannot be so easily separated, as speech fluency loaded onto both comprehension and production factors. In addition, these two factors could not be distinctively localized onto ventral and dorsal streams. Accordingly, numerous clinical studies, using different methods of analysis, have linked damage of the AF to deficits of various language faculties: speech fluency (Bates et al., 2003; Marchina et al., 2011; Fridriksson et al., 2013; Wang et al., 2013; Hope et al., 2016), informativeness of spontaneous speech (Marchina et al., 2011), sentence production (Wilson et al., 2011; Ivanova et al., 2016), naming (Wang et al., 2013; Geva et al., 2015; Hope et al., 2016; Ivanova et al., 2016), repetition (Breier et al., 2008; Kümmerer et al., 2013; Geva et al., 2015), and comprehension at the word and sentence level (Breier et al., 2008; Geva et al., 2015; Ivanova et al., 2016). Some studies have shown that the AF plays a role in comprehension of sentences with complex syntactic structures and high verbal-working memory demands (Dronkers et al., 2004; Wilson et al., 2011; Meyer et al., 2014). Obviously, the clear-cut dichotomy between dorsal and ventral streams proposed within different dorsal-ventral models of language processing does not stand up to empirical neuropsychological evidence. Further, speech arrest and anomic errors, particularly phonemic paraphasias, are observed when the AF is stimulated during awake surgery (Duffau et al., 2002; Bello et al., 2008; Maldonado et al., 2011). Language therapy for production deficits has been demonstrated to lead to changes in the integrity of the AF in the lesioned hemisphere (Breier et al., 2011; van Hees et al., 2014). Additionally, the AF is pivotal to language development (Bernal and Ardila, 2009; Perani et al., 2011; López-Barroso et al., 2013; Yeatman et al., 2013). Although there are single reports of excellent language recovery following disruption of the AF (Gyu and Ho, 2011; Chernoff et al., 2020) and a few studies demonstrating no clear relationship between damage to the AF and residual language abilities (Meier et al., 2019; Yu et al., 2019), the bulk of evidence strongly suggests that the AF is critical for language. However, given the breadth of its language associations, what remains to be determined is its specific functional contribution to various language capacities.

In addition to delineating the role of the AF in the left hemisphere, limited reports have indicated that this tract in the right hemisphere may play a compensatory role for language processing. First, Schlaug et al. (2009) showed that Melodic Intonation Therapy led to an increase in volume of the right AF in a group of chronic aphasia patients and hypothesized that these changes in the contralesional hemisphere supported long-term recovery. In another study, the volume of the long segment of the AF in the contralesional (right) hemisphere as measured at 2 weeks post-onset was predictive of language outcome at 6 months as indicated by a general measure of aphasia severity (Forkel et al., 2014). However, other studies failed to demonstrate a similar pattern between residual language abilities and right hemisphere tracts (Geva et al., 2015; Ivanova et al., 2016; Meier et al., 2019). Future studies are needed to further elucidate the contribution of the AF segments in the contralesional hemisphere to language recovery.

Part of the confusion regarding the functional role of the AF in language processing is most likely due to its undefined anatomy. Most studies to date still consider the AF as one indivisible uni-functional entity in accordance with classical language models (Wernicke, 1874; Geschwind, 1970). Recent research, however, has highlighted the importance of examining the functional significance of tract segments (rather than whole tracts), demonstrating that the integrity of the posterior portion of the AF was associated with both language production and comprehension deficits, while the anterior portion was related solely to production deficits (Ivanova et al., 2016). Further, different anatomical models of the AF have been recently suggested. Catani et al. (2005) proposed a 3-segment model of the AF, with the direct (long) branch of the pathway connecting Broca’s territory with Wernicke’s territory and likely supporting phonological processing. Two shorter, indirect branches connect inferior parietal areas (termed “Geschwind’s territory”) with Broca’s area (anterior segment) and Wernicke’s area (posterior segment) and were thought to support lexical-semantic processing. Only one study to date has utilized this model to explore the differential role of the AF segments in different language processes in aphasia. Forkel et al. (2020) in a study of individuals with primary progressive aphasia demonstrated the contributions of the posterior segment to repetition and the anterior segment to speech rate.

While the 3-segment model is the most common one, other models of the tract have been proposed as well. For example, Glasser and Rilling (2008) advanced a 2-segment model of the AF complex, proposing two distinct branches in the tract connecting temporal and frontal areas. According to their view, the AF stemming from the posterior superior temporal gyrus (STG; BA 22/Wernicke’s area) is the phonological pathway, while the second part of the AF branching inferior to the superior temporal sulcus in the posterior part of the middle temporal gyrus (MTG; BAs 21 and 37) is involved in lexical-semantic processing and has a pronounced left-ward asymmetry. Original support for this model comes from data on healthy controls and mapping of results of fMRI studies on phonological and lexical-semantic processing (Glasser and Rilling, 2008). Similarly, Friederici and Gierhan (2013) outlined two distinct branches within the AF complex, although attributed different functionality to them. The branch of the AF connecting the STG to Broca’s area (BA 44, pars opercularis) was proposed to be involved in complex syntactic processing, and the branch connecting STG/MTG to premotor cortex, in repetition. To the best of our knowledge, the functional distinctions proposed in these models have never before been directly tested in stroke survivors with language deficits. Also, surprisingly, none of these models have been previously evaluated with more advanced tractography algorithms relying on High Angular Resolution Diffusion Imaging (HARDI), despite previous work that has highlighted the differences in tract reconstruction based on different tracking algorithms, showing limitations in original tensor-based tractography for determining tract functionality (Li et al., 2013; Auriat et al., 2015; Ivanova et al., 2020).

Another related factor contributing to diverse findings might be the use of different methods and metrics to infer tract damage and delineate its specific role. Lesion load (e.g., Marchina et al., 2011; Wang et al., 2013), results of voxel-based lesion symptom mapping overlapped with standardized white matter atlases (e.g., Kümmerer et al., 2013; Corbetta et al., 2015), probability of disconnection (e.g., Salvalaggio et al., 2020), indices of microstructural integrity (e.g., Grossman et al., 2013; Ivanova et al., 2016), and residual tract volume (e.g., Forkel et al., 2014) have all been used as proxies to estimate the extent of tract damage. However, limited research suggests that these indices might not necessarily measure similar underlying properties (Hope et al., 2016; Forkel and Catani, 2018). Furthermore, the atlas-based approaches inherently cannot account for individual variability in premorbid tract configuration and subsequent tract damage (for more on limitations in application of atlas-based methods to clinical data see Forkel and Catani, 2018). Beyond that, cortical damage to adjacent areas has rarely been accounted for in evaluation of the AF’s role in language processing (for notable exceptions, see Griffis et al., 2017; Gajardo-Vidal et al., 2021). Finally, investigations often focus on a select number of language abilities, rather than evaluating language deficits in aphasia comprehensively, possibly obfuscating some of the brain-behavior relationships. Many of the limitations discussed above will be addressed in the present study.

Given the importance of the AF complex for language processing and outstanding questions concerning its anatomy and function, the overarching goal of the current study is to provide a comprehensive functional evaluation of the AF complex in both ipsilesional and contralesional hemispheres in a cohort of individuals with chronic post-stroke aphasia. For the first time, both the model by Catani et al. (2005) and the model by Glasser and Rilling (2008) were comprehensively evaluated in both hemispheres using an advanced tractography algorithm based on HARDI-data. Specifically, the aims of the study were to:

(1) Explore whether damage to different segments of the AF (defined according to the two different anatomical models) contributes differentially to language processing in individuals with chronic post-stroke aphasia.
(2) Investigate whether damage to the AF is predictive of language impairment beyond damage to known cortical language regions.
(3) Establish whether variations in the AF in the right (contralesional) hemisphere contribute to language outcomes in aphasia.

## Materials and Methods

### Participants

For years, the Center for Aphasia and Related Disorders at VANCHCS has been seeing stroke patients who complain of speech and language deficits. Those with left hemisphere lesions who participate in research at the Center undergo neuroimaging and comprehensive language testing. In the last several years, a HARDI-diffusion-weighted imaging sequence has been added to our scanning protocol, yielding a group of thirty-three successively scanned participants with aphasia (PWA; 24 males, 9 females) following a left hemisphere stroke (M_age_ = 63.7 1.4 years, from 40 to 83 years of age). All participants except 3 were strongly right-handed based on the Edinburgh Handedness Inventory (Oldfield, 1971), with the other 3 participants reporting a right-hand preference but some ambidexterity. All participants had native-like proficiency in English prior to their stroke. All participants had a single stroke, except for three individuals with small (<2 cm) asymptomatic secondary events, with the most recent incident being no less than 2 months prior to testing and scanning (M_time post–onset_ = 96.693.1 months). Patients in this sample presented with a wide range of speech and language deficits, some performing within normal limits on the WAB, but still complaining of residual naming and/or comprehension deficits (see “Results” section for more information). All patients signed IRB-approved consent forms and were tested in accordance with the Helsinki Declaration.

### Language Assessment

The Western Aphasia Battery (WAB; Kertesz, 1982, 2007) was administered to evaluate general language abilities. Participants were assessed with the 10 main language subtests, which cluster into Fluency, Information Content, Repetition, Naming, and Comprehension. Scores from these subtests comprise the WAB Aphasia Quotient (AQ), a general measure of aphasia severity.

Given that the AF has been most consistently related to naming and language comprehension abilities, participants were also assessed with two specific tests targeting these linguistic domains. To evaluate naming deficits, the short form of the Boston Naming Test (BNT; Kaplan et al., 2001) was used. This test measures word retrieval abilities by having participants name black and white line drawings, with targets varying in word frequency. On this test, phonemic errors are counted as correct, thus providing a more accurate index of underlying lexical-semantic abilities than the WAB naming subtest, which deducts points for phonemic errors.

The Curtiss-Yamada Comprehensive Language Evaluation-Receptive (CYCLE-R; Curtiss and Yamada, 1988) was administered for the assessment of language comprehension deficits at the sentence level. In this test, participants hear a sentence and are presented with three- or four-picture arrays, in which they have to select the target picture corresponding to the sentence. The test is particularly sensitive to deficits in syntactic processing, as it uses a very limited vocabulary while assessing comprehension of sentence structures with varying levels of difficulty. Twelve CYCLE-R subtests were grouped into three categories: Simple (simple sentence structure), Word Order (noncanonical word order), and Complex (multi clause relatives) (see Table 1 for subtest grouping). The Simple group contained the most unmarked type of sentence structure: simple intransitive and transitive, positive active declaratives with basic noun phrases consisting only of a determiner and a noun. In these sentences, there is either no ambiguity of role assignment or the roles are assigned according to canonical word order. The Word Order group included structures with noncanonical word order, that, unlike simple declarative sentences, require the assignment of animate referents to distinct thematic roles using basic morphosyntactic sentence structure. Their proper comprehension requires the identification of base grammatical structures either through the determination of morphological cues or word order information followed by thematic role assignment. The Complex group included sentences with various morphosyntactic structures that all involve relative clauses and so require the mapping of a noun phrase referent to a role in the main clause as well as a role in the embedded clause. In addition, they require a certain degree of maintenance and manipulation of syntactic and/or semantic information, particularly manipulating the components of complex sentence structures. One participant was not assessed with the CYCLE-R.

**TABLE 1:** Grouping of the Curtiss-Yamada Comprehensive Language Evaluation-Receptive (CYCLE-R) subtests.

Group	Subtest	Example
Simple	Possession	*The clown has a balloon.*
	Simple Declaratives	*The girl is sitting.*
	Active Voice Order	*The girl is pushing the boy.*
Word Order	Passive Voice Order I	*The dog is being pulled.*
	Passive Voice Order II	*The boy is being chased by the girl.*
	Object Clefting	*It’s the clown that the girl chases.*
	Negative Passive	*The girl is not being led by the boy.*
Complex	Subject Relatives	*The boy who is pulling the girl is mad.*
	Object Relatives	*The girl is chasing the clown who is big.*
	Double Embedding	*The clown that is big has the balloon that is red.*
	Object Relatives with Relativized Object	*The girl is kissing the boy that the clown is hugging.*
	Relative Pronouns with Double Function	*The girl who the boy is pushing is happy.*

### Brain Imaging

#### Data Acquisition

Brain imaging data were acquired on a Siemens Magnetom Verio 3T MRI scanner using a 12-channel head coil. High resolution structural data was acquired using a 3D T1-weighted MPRAGE protocol with 1 mm isotropic voxel resolution: TR = 2400 ms, TE = 3.16 ms, TI = 1000 ms, flip angle = 8°, FOV = 256 mm, imaging matrix = 256 × 256, acquisition time = 4.5 min. FLAIR and fast spin echo T2-weighted images were also acquired with the default Siemens pulse sequences to improve segmentation of brain lesions. Diffusion-weighted imaging (DWI) sequences were collected with the following parameters: TR = 17600 ms, TE = 93.6 ms, flip angle = 90°, *b* = 2000 s/mm^2^, 64 directions, 10 b0, FOV = 240 mm, voxel size 2 × 2 × 2 mm, 65 axial slices, bandwidth = 1812 Hz/voxel, and GRAPPA factor = 2.

#### Lesion Reconstructions

The participants’ lesions were traced directly onto the patient’s native T1-weighted images using MRIcro/MRIcron software (Rorden and Brett, 2000). During this procedure, the T2-weighted and FLAIR images were co-registered to the T1 images to verify lesion boundaries. Then the T1 image and subsequently the binary lesion mask were normalized to an MNI template using a modified version of the unified segmentation/normalization algorithm implemented in SPM8 with cost function masking of the lesion (“Seg” toolbox in the SPM8 distribution; Crinion et al., 2007). This algorithm was customized to optimize normalization of deep white matter and ventricles by using an age relevant template and by additionally incorporating a head model (Turken et al., 2010; Ivanova et al., 2016), providing a tighter fit to the template space without distorting overall brain anatomy (Crinion et al., 2007).

We then estimated lesion load to three cortical language areas: frontal, temporal, and parietal cortex. These areas were defined based on the Harvard-Oxford Cortical Structural Atlas in FSL (Desikan et al., 2006; Jenkinson et al., 2012). The frontal language area was defined as the inferior frontal gyrus, including both pars opercularis and pars triangularis, thresholded at 20%. The temporal language area was based on the superior temporal gyrus posterior division and the middle temporal gyrus posterior division, thresholded at 20%. The parietal language area included the angular gyrus and the supramarginal gyrus (both the anterior and the posterior divisions), again thresholded at 20%. These areas were chosen because they are commonly implicated in language processing and their damage typically leads to distinct aphasia types (Dronkers and Baldo, 2010; Turken and Dronkers, 2011; Duffau et al., 2014; Henseler et al., 2014; Dronkers et al., 2015; Dragoy et al., 2017). For each participant, lesion load for each area (i.e., the proportion of the area covered by the lesion) was calculated.

#### DWI Data Processing

Diffusion-weighted imaging data were first preprocessed using a fieldmap correction for susceptibility induced distortions (FSL ver. 5.09, Jenkinson et al., 2012), followed by movement and eddy current corrections (ExploreDTI ver. 4.8.6, Leemans et al., 2009). Next, HARDI deterministic tractography based on constrained spherical deconvolution was done using these parameters: ALFA – 1.8, iterations – 300, n – 0.002, r – 15, ABS threshold – 0.003, step size (mm) – 0.5, angle threshold – 35, minimal length (mm) – 50 (StarTrack beta version, www.mr-startrack.com, Dell’Acqua et al., 2013).

#### Manual Tract Segmentation

**FIGURE 1:**
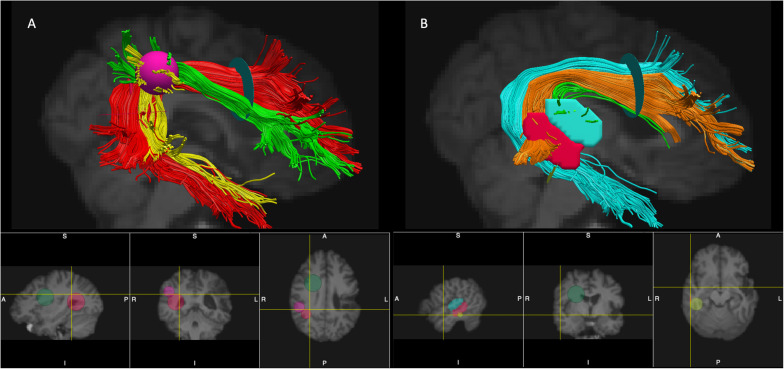
Placement of ROIs and segmentation of the AF (the right hemisphere is shown). Panel **(A)** - segmentation according to the modified Catani model: AF long – red, AF anterior – green, and AF posterior – yellow. Panel **(B)** – segmentation according to the modified Glasser and Rilling model: AF-STG – green, AF-MTG- orange, and AF Temporal Pole – cyan. The ROIs used for segmentations of the tracts: Frontal ROI – light green 2D disk, Temporal ROI – red 2D disk, Parietal ROI – pink 3D sphere, STG ROI – cyan manually drawn region, MTG ROI – red manually drawn region, Temporal Pole ROI – yellow 2D disk (see text for more details per reconstruction criteria). Note that 2D disks used as ROIs (Frontal ROI, Temporal ROI, Temporal Pole ROI) are visualized in TrackVis as 2D spheres on the bottom panels.

*In vivo* manual tract dissections of the AF from whole brain tractograms in native space were completed (TrackVis ver. 0.6.1, Wang et al., 2007). AZ and MI performed the reconstructions together according to the criteria outlined below, and reconstructions were then reviewed and revised together with ND. The AF in the left and right hemispheres were segmented according to two different anatomical models with modifications: the Catani 3-segment model (Catani et al., 2005) and the Glasser and Rilling (2008) 2-segment model. See Figure 1 for ROI placement and an example of AF segmentation in the right hemisphere.

##### Modified 3-segment Catani model

We reconstructed a modified version of the AF complex proposed by Catani et al. (2005). Given that the cortical terminations of the AF in the frontal and temporal lobes remain undetermined (and greatly influenced by the underlying tracking algorithm), we did not specifically limit the terminations of the tract within those lobes. Accordingly, instead of defining the three branches of the AF by their cortical terminations as was done previously (Catani et al., 2005; Forkel et al., 2014), we based our segmentation largely on the connections between different lobes (see Mandonnet et al., 2018 for a similar approach to tract classification) and used the following ROIs to segment the three branches: a Frontal ROI – a 2D disk placed on the coronal slice at the entrance to the frontal lobe (anterior to the central sulcus), a Temporal ROI – a 2D disk placed on the axial slice at the entrance to the temporal lobe (below the Sylvian fissure), and a Parietal ROI – a 3D sphere placed tangent to the inferior parietal cortex. The size of the disks and spheres varied slightly between participants depending on brain size. The three segments of the AF in both hemispheres were extracted according to the following criteria.

• **AF long** – A tract connecting the temporal and the frontal areas dorsally was defined by the Frontal and Temporal ROIs. During the reconstruction, the ROIs were expanded to make sure all the fibers were included. Looping fibers and fibers going between the temporal and the frontal lobe ventrally through the external capsule were excluded.

• **AF anterior** – A tract connecting frontal regions with inferior parietal areas was defined by the Frontal and Parietal ROIs. Additionally, the Temporal ROI was used as a NOT region, so that only fibers extending dorsally from the frontal to the inferior parietal areas were captured, but not any fibers continuing to the temporal lobe. We also ensured that the anterior short segment passed directly adjacent and laterally to the long segment of the AF, while fibers passing more superiorly to the AF long tract, as well as those separated from it by a gap were excluded.

• **AF posterior** – A tract connecting inferior parietal regions with the temporal lobes, was defined by the Parietal and Temporal ROIs. Additionally, the Frontal ROI was used as a NOT region, so that only fibers extending laterally from the inferior parietal areas to the temporal cortex were captured, while excluding any fibers continuing to the frontal lobe. We also confirmed that this posterior short segment passed laterally to the long segment of the AF.

##### Modified Glasser and Rilling model

We reconstructed a modified version of the AF complex according to the Glasser and Rilling (2008) model. Essentially this model provided a further subdivision of the long segment of the AF within the Catani model with different branches of the AF distinguished by their terminations in the temporal lobe. In addition to the two segments identified in the original Glasser and Rilling model – the AF terminating in the posterior superior temporal gyrus (STG) and the posterior middle temporal gyrus (MTG) – we also observed that a substantial part of the long segment was extending to the temporal pole and was not a part of the two original segments. Accordingly, in our reconstructions we made a post-hoc decision to include it as a third branch extending into the temporal pole. The three segments were reconstructed according to the following criteria.

• **AF-STG** – fibers extending between the posterior STG and the frontal lobe, defined by a manually drawn ROI placed in the white matter underneath the posterior STG cortex on the sagittal slices and the Frontal ROI (the same one as used above for segmentations of the AF according to the Catani model). The size of the STG ROI depended on the individual’s brain anatomy.

• **AF-MTG** – fibers extending between the posterior MTG and the frontal lobe, defined by a manually drawn ROI placed in the white matter underneath the posterior MTG cortex on the sagittal slices and the Frontal ROI. Again, the size of the MTG ROI depended on the individual’s brain anatomy.

• **AF temporal pole** – fibers extending from the frontal lobe all the way to the anterior part of the temporal lobe, including the temporal pole, defined by an Temporal Pole ROI and the Frontal ROI. The Temporal Pole ROI was a 2D disk placed on the coronal slice in the middle of the temporal lobe behind the STG and MTG ROIs. The AF temporal pole branch did not include fibers terminating in the posterior STG or MTG cortex.

For each segmented tract, we extracted tract volume and mean Hindrance Modulated Orientation Anisotropy (HMOA, Dell’Acqua et al., 2013). HMOA is a measure of tract integrity reflective of the amount of the diffusion for a given fiber orientation. It is similar to the more traditional fractional anisotropy, but instead of being voxel based, it is tract based. For analysis, tract volume measures were normalized by the participant’s hemisphere volume to account for variations in head size. We used these two tract metrics – normalized volume and HMOA – in all of the analyses. In those instances when segments of the AF had been destroyed by the stroke and could not be reconstructed, zeroes were imputed for respective tract volume and HMOA values.

### Data Analysis

First, we correlated tract metrics (normalized volume and HMOA) of all tract segments for both hemispheres with language measures while taking relevant demographic variables and lesion volume into account. Beyond outlined tract segments, we also included overall AF volume (combination of the 3 segments within the Catani model, labeled “AF all”) in the correlation analysis to evaluate the overall role of the AF in language. To account for multiple comparisons, we adjusted the significance level in the correlation analyses for each tract with the False Discovery Rate (FDR) correction (Benjamini and Hochberg, 1995). Second, we performed a series of hierarchical regression analyses to determine whether residual tract integrity in the left hemisphere contributed significantly to language performance beyond damage to known cortical language areas. The analyses were run in IBM SPSS ver. 23.

## Results

### Descriptive Statistics

Prior to running any analyses, we screened our behavioral and neuroimaging data for outliers. For behavioral data, we looked at WAB AQ scores (as a general measure of language severity) and for neural data, we examined lesion volume. Boxplots of these two variables are presented in the Supplementary Figure A1. One outlier was present in both indices: the same individual had the largest lesion volume (380430 voxels) and the smallest WAB AQ (22.8) and was 3 SDs from the mean for both indices. For this participant, none of the AF segments in the left hemisphere could be reconstructed. Accordingly, this participant was eliminated from all further analyses.

For the remaining 32 participants descriptive statistics for language scores and tract metrics are presented in Tables 2, 3, respectively. PWA had language deficits ranging from severe to mild, with the milder patients still demonstrating residual naming and/or comprehension deficits, as evidenced by lower naming scores and CYCLE-R scores. Table 3 contains Pearson correlations between normalized volume and HMOA for each tract. These correlations showed that these two tract metrics were significantly related within each segment, especially for the left hemisphere tracts, where the relationship was bolstered by tract damage. For results of correlational analyses between tract metrics across different segments of the AF see Supplementary Tables 1A, 2A. Paired *t*-tests showed that volume and HMOA measures in the left (perilesional) hemisphere were significantly lower compared to the right (contralesional) hemisphere for all AF segments (*p* < 0.001), except for HMOA of AF posterior (*p* = 0.066).

**TABLE 2:** Descriptive statistics for the language scores (*n* = 32*).

	Language test	Max score possible	*M*	*SD*	Range
WAB	Fluency	10	8.00	2.24	2-10
	Information content	10	8.69	1.86	3-10
	Repetition	10	8.01	2.19	1.8-10
	Naming	10	7.78	2.40	1.6-10
	Comprehension	10	8.83	1.31	6.4-10
	AQ^†^	100	82.60	17.42	39.6-99.8
BNT		15	10.29	4.45	0-15
CYCLE	Simple	5	4.71	0.45	3.67-5
	Word Order	5	3.47	1.22	0.5-5
	Complex	5	3.30	1.14	1.2-5

*CYCLE scores are presented for n = 31, as one participant did not complete the test. ^†^AQ > 93.8 is considered performance within normal limits, though patients performing in that range still continue to report minor language deficits.

**TABLE 3:** Descriptive statistics [Mean (*SD*)] for the tract metrics and correlations (Pearson’s r) between them (*n* = 32).

Tract metric	AF long	AF anterior	AF posterior	AF-STG	AF-MTG	AF-temporal pole
	
	Left hemisphere					
Volume (in mm^3^)	13637 (12773)	5709 (5205)	6578 (6706)	1765 (2518)	4183 (5309)	4513 (5587)
HMOA	0.0109 (0.0068)	0.0099 (0.0058)	0.0091 (0.0058)	0.0051 (0.0065)	0.0075 (0.0081)	0.0087 (0.0071)
Corr.: Normalized Volume × HMOA	0.79***	0.62***	0.62***	0.87***	0.88***	0.71***

	**Right hemisphere**					

Volume (mm^3^)	33021 (8690)	18183 (7066)	7228 (4285)	6178 (2762)	13674 (5532)	11356 (5711)
HMOA	0.0164 (0.0016)	0.0172 (0.002)	0.0112 (0.0016)	0.0122 (0.0014)	0.0165 (0.0019)	0.0151 (0.002)
Corr.: Normalized Volume × HMOA	0.56***	0.39*	0.43*	0.59***	0.62***	0.36*

*p < 0.05 and ***p < 0.001.

**FIGURE 2:**
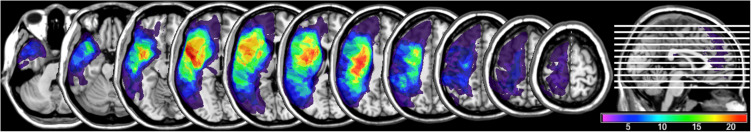
Lesion overlay map (*n* = 32) demonstrating overlap across participants’ lesions, with brighter colors indicating greater number of patients having a lesion in each voxel (ranging from a minimum of one participant’s lesion in a voxel and a maximum of 21).

**FIGURE 3:**
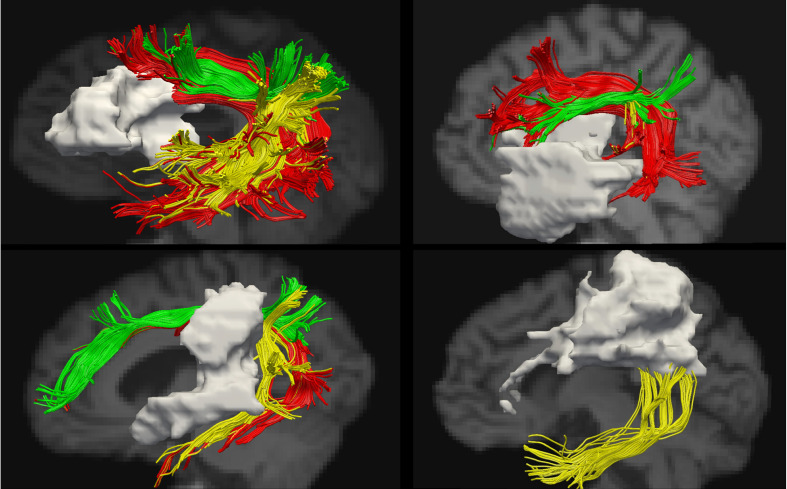
Examples of reconstructions of the AF segments in the left hemisphere. The lesion is shown in gray. Segmentation according to the modified Catani model: AF long – red, AF anterior – green, and AF posterior – yellow.

See Figure 2 for lesion overlays and Figure 3 for example of reconstructions of the left AF. As can be clearly seen in Figure 3, the reconstructed tracts varied significantly depending on the participant’s lesion size and location. See also Supplementary Table 3A for information on which AF segments were constructed in the left hemisphere for each participant.

### Correlational Analysis With Language Measures

First, we established which demographic (age, gender) and stroke-related (time post-onset, lesion volume) covariates had an impact on test performance and/or tract indices based on Pearson correlations and independent-samples *t*-test (for gender). Age and time post-onset were not significantly related to any language or tract indices (*p* > 0.05). Significant differences in performance based on gender were observed for the auditory comprehension subtests of the WAB and the CYCLE-R, with female participants obtaining higher scores (*p* < 0.05). Lesion volume was significantly negatively related to WAB and CYCLE-R subtest scores; it also correlated strongly with both tract metrics (*p* < 0.05). Thus, to conserve statistical power, based on empirical evaluation of association patterns we included gender and lesion volume as covariates in the correlational analyses below. Additionally, given the substantial variability in post-onset times amongst participants and the fact the time post-onset has been a factor in other studies (Saur et al., 2006), we felt it was necessary to add it as covariate in the partial correlational analysis below, despite it not having a detectable relationship with either the language scores or the tract metrics.

The results of this partial correlational analysis are presented in Table 4 (for analysis accounting for effect of gender only see Supplementary Table 4A). The overall AF volume was related to naming, comprehension, and aphasia severity. Tract volume of the AF long was only related to auditory language comprehension, particularly for more complex sentences, and naming on the BNT. However, these correlations did not survive the FDR correction for multiple comparisons. With the correction only the associations between the volume of the AF posterior and language scores remained significant with the correction, specifically the volume of the AF posterior was related to a number of lexical-semantic and syntactic language abilities, including auditory comprehension, naming, and repetition. The AF anterior was not associated with any language scores. All three branches of the long segment (from the modified Glasser and Rilling model) were related to naming as measured by the BNT, but after correcting for multiple comparisons only the correlation for the AF temporal pole branch remained significant. These three branches were not related to any of the other language measures.

**TABLE 4:**
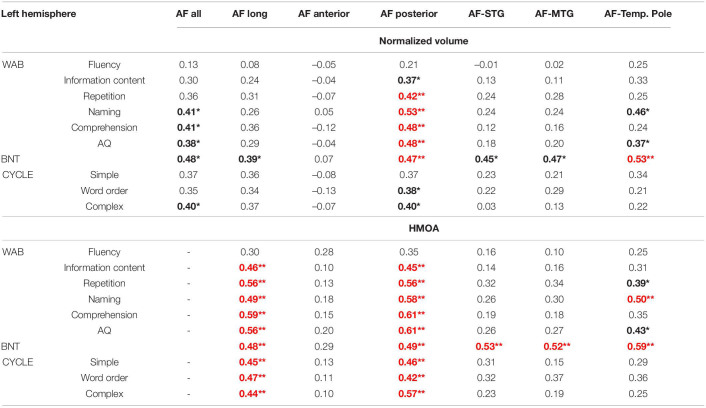
Results of partial correlational analysis (Pearson’s r) between tract metrics of the left arcuate fasciculus (AF) and language indices accounting for gender, time post-onset and lesion volume.

*p < 0.05, uncorrected (in the color version correlations significant at this level are shown in black boldface), ** p < 0.05, FDR corrected (in the color version correlations significant at this level are shown in red boldface).

The observed relationship between HMOA and language scores further extended these findings with most of the correlations surviving the stringent corrections for multiple comparisons. AF long and AF posterior were related to most of the language measures, while all three branches of the AF within the Glasser and Rilling model were again associated with naming.

In contrast to the left AF tracts, the right hemisphere tracts did not show consistent or strong relationships with language measures, and the few significant correlations did not survive the corrections for multiple comparisons (for results of this correlational analysis see Supplementary Table 5A).

### Contribution of the AF Beyond Known Cortical Language Areas

Next, we ran a series of multiple regression analyses to determine whether residual tract integrity of the AF complex in the left hemisphere was related to language outcome beyond damage to specific frontal and temporal cortical language areas. In the first step of this hierarchical regression analysis, lesion load to frontal, temporal, and parietal language areas along with gender and time post-onset were entered as covariates. Lesion volume was not entered into the analysis since it correlated strongly with lesion load to language areas. In the second step, either normalized volume (Step 2a) or HMOA (Step 2b) of the three segments of the left AF according to the Catani model were entered into the analysis using the forward procedure (probability to enter *p* < 0.05). Results of the regression analyses for language scores where the significant role of tracts was observed beyond that of cortical language areas are presented in Table 5. Overall, beyond contribution of cortical language areas, the residual volume of the long segment was related to naming abilities, while HMOA measurements highlighted the complimentary importance of the posterior segment for lexical-semantic processing and the anterior segment for fluency and naming.

**TABLE 5:** Results of multiple hierarchical regression analysis investigating whether the AF tracts in the left hemisphere (according to the modified Catani model) are related to language scores beyond damage to specific frontal, temporal, and parietal cortical language areas.

Model	Fluency		Information content		Repetition		Naming		Comprehension		WAB AQ		BNT		CYCLE Simple		CYCLE Word Order		CYCLE Complex	
										
	*Adj.*	*Sig.*	*Adj.*	*Sig.*	*Adj.*	*Sig.*	*Adj.*	*Sig.*	*Adj.*	*Sig.*	*Adj.*	*Sig.*	*Adj.*	*Sig.*	*Adj.*	*Sig.*	*Adj.*	*Sig.*	*Adj.*	*Sig.*
	*R*^2^	*F Change*	*R*^2^	*F Change*	*R*^2^	*F Change*	*R*^2^	*F Change*	*R*^2^	*F Change*	*R*^2^	*F Change*	*R*^2^	*F Change*	*R*^2^	*F Change*	*R*^2^	*F Change*	*R*^2^	*F Change*
Step 1 – Specific cortical areas (temporal, frontal, parietal regions), gender and time post-onset	0.319	0.011	0.220	0.045	0.574	<0.001	0.445	0.001	0.407	0.002	0.509	<0.001	0.357	0.006	0.304	0.013	0.299	0.015	0.301	0.014
Step 2a – Tract normalized volume	-	n.s.	-	n.s.	-	n.s.	-	n.s.	-	n.s.	-	n.s.	0.468	0.02	-	n.s.	-	n.s.	-	n.s.
Significant predictors	-		-		-		-		-		-		AF long		-		-		-	
Step 2b – Tract HMOA	0.461	0.011	-	n.s.	-	n.s.	0.663	0.008	0.480	0.044	0.635	0.028	0.582	0.005	-	n.s.	-	n.s.	0.388	0.043
Significant predictors	AF anterior		-		-		AF anterior, AF posterior		AF posterior		AF anterior, AF posterior		AF anterior AF posterior		-		-		AF posterior	

*n.s., not significant.*

Next, we conducted the same analysis using the Glasser and Rilling AF segmentation. Here, only significant contributions of the AF temporal pole normalized volume to naming scores on the WAB (Adj. *R*^2^ = 0.530, Sig. *F* Change = 0.028) and BNT (Adj. *R*^2^ = 0.493, Sig. *F* Change = 0.01), as well as the AF temporal pole HMOA to naming scores on the BNT (Adj. *R*^2^ = 0.497, Sig. *F* Change = 0.009) could be established. These findings underscore the role of the long-range temporal-frontal connections, particularly those extending to the anterior part of the temporal lobe, in naming ability.

## Discussion

The current study explored how stroke-induced damage to the AF impacts language processing abilities in individuals with chronic post-stroke aphasia. We segmented the AF in both hemispheres using two anatomical models: the modified 3-segment model by Catani et al. (2005) and a modified version of the 2-segment model by Glasser and Rilling (2008) that additionally incorporated an anterior temporal lobe extension. Our results indicated that the left AF, particularly its posterior segment, is critical for a number of language processes, including comprehension, repetition and naming.

### Relationship Between AF Integrity in the Left Hemisphere and Language Abilities

The AF is a large, dense tract that connects numerous frontal and temporal language regions. In this study, reconstructions of the AF included connections not described in existing models. Specifically, when using the segmentation according to the Catani et al. (2005) model, the different segments were defined by their connectivity profiles between different lobes, so as not to limit tract segmentations to circumscribed predefined cortical areas (see Mandonnet et al., 2018 for a similar approach to tract descriptions). Given the extensive variability observed in individual tract reconstructions (Catani et al., 2005; Auriat et al., 2015; Wang et al., 2016) and that this 3-segment AF model has not been previously used with more advanced tractography algorithm, we felt that we would be excluding potentially crucial connections by basing our reconstructions on cortical regions derived from tensor models. Further, to capture all the fibers in the AF long segment, the Glasser and Rilling (2008) model was supplemented post-hoc by an additional branch extending into the temporal pole. Again, because previously this model was not used with advanced tractography algorithms this subdivision might have gone undetected.

In the current study, damage to the AF was associated with a broad range of aphasic language deficits. As expected, the correlations between tract and language metrics significantly diminished once lesion volume was taken into account, as lesion volume was strongly associated with both indices. With larger lesions, volume of long associative fibers is more likely to be reduced and, at the same time, more pronounced behavioral deficits are expected. Accordingly, similar to lesion symptom mapping analyses (Ivanova et al., 2021), investigations of tract functionality need to account for lesion volume effects to determine exclusive and differential contribution of tracts to observed deficits.

When the AF segments within the Catani et al. (2005) model were considered separately, a distinctive role of the AF segments was discerned. Microstructural integrity of the long segment of the left AF as measured by HMOA was associated strongly with informativeness of speech output, repetition, naming and comprehension, even after partialling out lesion volume, and effects of gender along with time post-onset. Further, the data demonstrated that the posterior segment of the left AF plays a vital role in language processing with both its volume and microstructural integrity strongly related to a broad range of lexical-semantic processes, including repetition, naming and comprehension abilities. In addition, we subdivided the long segment of the AF according to the Glasser and Rilling (2008) model depending on different terminations in the temporal lobe. No functional distinction between the long segments originating from MTG and STG was observed, and both, along with anterior temporal lobe extensions, were associated with lexical-semantic retrieval.

Current results indicated that HMOA is a sensitive measure of tract integrity reflecting important aspects of residual function. While the two tract measures, volume and HMOA, largely index similar properties, as shown by their high intercorrelations, particularly in the left hemisphere where the relationship is largely driven by the underlying stroke lesion, they are not identical. Volumetric measurements reflect how much of the tract is still left, while HMOA is more indicative of the integrity of the residual tract, approximating how well the remaining parts of the tract are able to function. For a comprehensive evaluation of structural connectivity these measures need to be used in tandem. Together, observed patterns of associations between both tract metrics and language scores are highly consistent with multiple previous studies on the functional role of the AF (Breier et al., 2008; Marchina et al., 2011; Wilson et al., 2011; Kümmerer et al., 2013; Wang et al., 2013; Dronkers et al., 2015; Geva et al., 2015; Hope et al., 2016; Ivanova et al., 2016), and show that, depending on how the tracts are segmented, different functional relationships can be established.

The current study was the first one to explore the functionality of the AF according to the Glasser and Rilling (2008) model. This model focuses specifically on the frontal-temporal pathway, so it only includes subdivisions of the AF long segment and does not involve any connections terminating in the parietal lobe. Interestingly, no clear distinction between STG and MTG branches of the long segment of the AF could be detected, contrary to what the 2-segment model of the AF postulates (Glasser and Rilling, 2008). All of the three branches, and particularly the anterior temporal lobe extension, contributed to residual naming ability. This lack of functional specificity possibly reflects the fact that due to their anatomical proximity, the three branches were often damaged together. High intercorrelations observed between volumes of the three branches (see Supplementary Table 1A) potentially support this explanation, although by looking at individual data (Supplementary Table 3A) we can see that there were a number of patients in whom the three branches were selectively identified, enabling us to explore their differential contributions. Alternatively, this lack of functional specificity between AF branches with different terminations in the temporal lobe might indicate that for language processing, global integrity of a direct connection between the temporal and the frontal lobes is more critical than specific tract terminations within the temporal lobe (see López-Barroso et al., 2013 for a similar argument). Future research with more variable damage in the temporal lobes is needed to tease apart these alternative interpretations.

Further, for the first time, the contribution of the AF to language processing was comprehensively investigated while damage to relevant cortical language areas was systematically taken into account. Beyond the contribution of these classical frontal, posterior temporal, and inferior parietal language areas, the volume of the long segment of the AF and particularly the AF temporal pole segment was an important predictor for lexical-semantic retrieval. With HMOA measurements of tract integrity, it was demonstrated that the anterior and posterior segments of the left AF are also important for overall language abilities. Specifically, AF anterior, similar to previous findings (Bates et al., 2003; Fridriksson et al., 2013; Wang et al., 2013; Gajardo-Vidal et al., 2021) is crucial for expressive language abilities, such as fluency and naming, while the posterior segment is important for both lexical-semantic processing (Geva et al., 2015; Ivanova et al., 2016) and comprehension (Wilson et al., 2011; Ivanova et al., 2016). These results reinforce the importance of examining structural disconnection using tractography to better understand language outcomes in aphasia, as these tracts continue to make a unique contribution to residual language functioning even when damage to cortical language areas is taken into account.

Overall, the current study highlighted that the contribution of the long segment of the AF to language might support more general language functions than specific ones (for a related argument see Dick and Tremblay, 2012), as residual tract volume and integrity were related to a number of language abilities. Also, the results indicate that the posterior segment of the AF makes a vital contribution to language processing, mirroring multiple previous studies (Ivanova et al., 2016; Griffis et al., 2017) that highlighted the crucial role that these posterior connections play in language. This is similar to another recent investigation of the functional role of the AF in language in individuals with aphasia, which found that the group with the damaged posterior segment of the AF obtained the lowest language scores (Yu et al., 2019). The crucial role of the posterior segment of the AF also reinforces a previously proposed notion that interlobar connectivity of posterior temporal cortex is vital to language processing (Turken and Dronkers, 2011).

### Role of Right Hemisphere Tracts

In the current study, we did not find any clear contribution of the right hemisphere AF segments to language performance. The overall pattern of correlations between tract metrics of the right AF and language scores was very sparse and not easily generalizable, with none of them surviving the FDR correction for multiple comparisons. This finding is consistent with previous stroke studies that also failed to find a coherent relationship between the right hemisphere AF and language (Geva et al., 2015; Ivanova et al., 2016; Meier et al., 2019). A potential exception is a study by Forkel et al. (2014), in which the volume of the right AF as measured at two weeks post stroke robustly predicted the level of language recovery at 6 months post stroke. However, our study did not measure recovery over time, but rather correlated AF volume/HMOA directly to individuals’ current language performance in the chronic stage of stroke across highly heterogenous post-onset times. It is also possible that the difference in results was impacted by the particular tractography algorithm employed: tensor-based versus HARDI (see Li et al., 2013 and Ivanova et al., 2020 on comparing reconstructions of the AF based on different algorithms). In other clinical populations, such as patients with brain tumors in the left hemisphere (Jehna et al., 2017) and children with developmental disorders (Paldino et al., 2016), it has been shown that a smaller AF in the right hemisphere leads to worse language outcomes. Specifically for tumor patients Jehna et al. (2017) showed descriptively that patients with fewer language deficits tended to have a symmetric AF or the one lateralized to the right, while those with pronounced language deficits more often had a left-lateralized posterior segment of the AF. Thus, larger AF on the right contributed to better language outcomes, however, only the comparison of the spontaneous speech metric was statistically significant between the two groups differing in AF laterality. Further work is needed to establish the role of the right (and left) AF in language recovery over time.

### Limitations

The current study had several limitations that should be addressed in future investigations. Although we used a number of specialized language tests such as the CYCLE and BNT, we relied on the WAB for a number of measures. Supplemental and more linguistically sensitive tests may be useful in further isolating the specific and unique roles of the AF and its segments in language processing.

In terms of tract reconstructions, we did not explore all possible segmentations of the AF. With our tractography algorithm it was not possible to segment the AF according to Friederici and Gierhan (2013) model. Future investigations are encouraged to explore this model as well. In addition, larger cohorts with more variability in lesion location might add to the differential functionality of the tracts. As stated previously, regarding the lack of clear tract-specific functional relationships within the Glasser and Rilling (2008) model, tracts that are located close together tend to often be damaged together, making it challenging to ascertain their unique functionality (or lack thereof). Thus, a cohort with smaller lesions and possibly different etiology (such as tumor patients) is required to explore this further and tease apart competing interpretations.

It is also possible that idiosyncratic reorganization and compensation in chronic stroke led to varied effects, particularly given the large variability in post-onset times and that many of the participants in the current study had only very mild residual language deficits. Even though we accounted for heterogenous post-onset times in our analyses, this variability might have masked differential effects at different stages of recovery, such as contribution of the right hemisphere areas and tracts in early stages post-stroke (e.g., Saur et al., 2006; Forkel et al., 2014). To better understand and dissociate these possible mechanisms, future work should attempt to map the contribution of white matter fiber pathways during different stages of recovery in a longitudinal study. Further, the role of tracts in the contralesional hemisphere should be explored using multimodal imaging in order to highlight both structural and functional connectivity and possible reorganization.

## Conclusion

In sum, our findings lend support to the broader role of the AF in language processing, with particular emphasis on comprehension and naming, and point to the posterior segment of this tract as being most crucial for supporting residual language abilities. The findings stand in contrast to the dorsal-ventral models of language processing that ascribe a specific role to the dorsal stream and AF in particular, limiting its role to repetition abilities and comprehension at the sentence level. Current results highlight the importance of considering additional tract segments that should also be incorporated into future models of language processing and underscore the importance of considering different anatomical subdivisions of given tracts.

## Data Availability Statement

The datasets presented in this article are not readily available: Research data are not shared per Department of Veteran Affairs privacy regulations. Requests to access the datasets should be directed to ND, dronkers@berkeley.edu.

## Ethics Statement

The studies involving human participants were reviewed and approved by the VA Northern California Health Care System IRB. The patients/participants provided their written informed consent to participate in this study.

## Author Contributions

MI contributed to conception, design of the study, developed the diffusion data processing pipeline, performed the statistical analysis, and wrote the original draft of the manuscript. AZ and MI performed the tractography and lesion segmentations, and subsequent data coding. AT developed the MRI scanning protocols and subsequent raw data processing algorithms and provided funding for the project. JB facilitated behavioral data collection and organized the database. ND contributed to conception, design of the study, funding acquisition, and oversaw execution of the project. All authors contributed to manuscript revision, read, and approved the submitted version.

## Conflict of Interest

The authors declare that the research was conducted in the absence of any commercial or financial relationships that could be construed as a potential conflict of interest.
