# Supplementary material for: Functional Contributions of the Arcuate Fasciculus to Language Processing
Source: Front Hum Neurosci. 2021 Jun 25;15:672665. doi: 10.3389/fnhum.2021.672665 (PMC8267805; doi:10.3389/fnhum.2021.672665)
Supplement: Supplementary file 1 [file Data_Sheet_1.pdf]

Appendix A.

Supplementary materials.

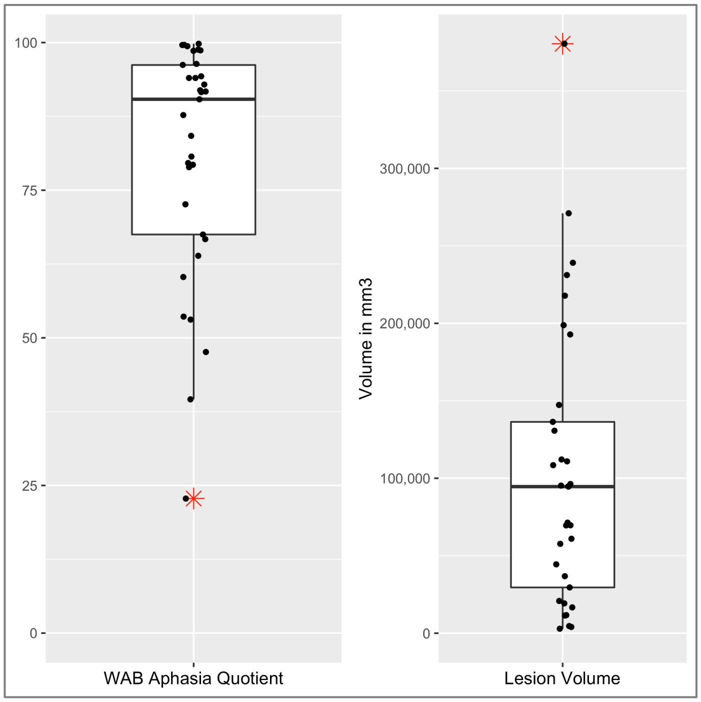

Figure 1A. Box plots of participants' WAB AQ and lesion volume. The outlier is marked with a red asterisk.

Table 1A. *Results of correlational analysis (Pearson's r) between tract metrics across different segments of the AF in the left hemisphere.*

Left hemisphere		AF long	AF anterior	AF posterior	AF-STG	AF-MTG	AF-Temporal Pole	AF long	AF anterior	AF posterior	AF-STG	AF-MTG	AF-Temporal Pole
		Volume						HMOA					
AF long	Volume	1											
AF anterior		.471**	1										
AF posterior		.402*	.163	1									
AF-STG		.733***	.356*	.208	1								
AF-MTG		.790***	.445*	.406*	.895***	1							
AF-Temporal Pole		.707***	.561***	.564***	.632***	.730***	1						
AF long	HMOA	.788***	.299	.455**	.531**	.598***	.554***	1					
AF anterior		.541***	.624***	.15	.403*	.478**	.458**	.520**	1				
AF posterior		.534**	.172	.616**	.243	.341	.378*	.668***	.212	1			
AF-STG		.806***	.417*	.357*	.873***	.904***	.769***	.592***	.508**	.335	1		
AF-MTG		.750***	.385*	.462**	.732***	.875***	.721***	.630***	.521**	.355*	.876***	1	
AF-Temporal Pole		.639***	.349*	.530**	.615***	.725***	.712***	.729***	.563***	.475**	.738***	.826***	1

Note. ***** $p < .05$, ****** $p < .01$, *** $p < .001$.

Table 2A. *Results of correlational analysis (Pearson's r) between tract metrics across different segments of the AF in the right hemisphere.*

Right hemisphere		AF long	AF anterior	AF posterior	AF-STG	AF-MTG	AF-Temporal Pole	AF long	AF anterior	AF posterior	AF-STG	AF-MTG	AF-Temporal Pole
		Volume						HMOA					
AF long	Volume	1											
AF anterior	Volume	.424*	1										
AF posterior	Volume	.092	.336	1									
AF-STG	Volume	.133	.181	.207	1								
AF-MTG	Volume	.672**	.380*	.352*	.313	1							
AF-Temporal Pole	Volume	.474**	.323	-.087	-.118	.274	1						
AF long	HMOA	.561**	.199	.231	.365*	.565***	.22	1					
AF anterior	HMOA	.3	.388*	.154	.538**	.285	.222	.444*	1				
AF posterior	HMOA	-.223	.173	.434*	.134	.08	-.15	.149	.099	1			
AF-STG	HMOA	.012	.365*	.442*	.591***	.258	-.295	.31	.472**	.377*	1		
AF-MTG	HMOA	.510**	.191	.246	.244	.615***	.192	.850***	.407*	.098	.371*	1	
AF-Temporal Pole	HMOA	.456**	.485**	.234	.415*	.487**	.359*	.753***	.537**	.243	.366*	.591***	1

Note. ***** $p < .05$, ****** $p < .01$, ******* $p < .001$.

Table 3A. *Information regarding which AF segments were reconstructable in the left hemisphere for each participant.*

Participant #	AF long	AF anterior	AF posterior	AF-STG	AF-MTG	AF-Temporal Pole
1	yes	yes	yes	no	yes	yes
2	no	yes	no	no	no	no
3	yes	yes	yes	yes	yes	yes
4	yes	yes	yes	yes	yes	yes
5	no	no	no	no	no	no
6	yes	yes	yes	no	no	yes
7	yes	yes	yes	no	yes	yes
8	yes	yes	yes	yes	yes	yes
9	no	yes	no	no	no	no
10	no	no	no	no	no	no
11	yes	yes	yes	yes	yes	yes
12	yes	yes	yes	yes	yes	yes
13	yes	yes	yes	yes	yes	yes
14	yes	yes	no	no	yes	yes
15	yes	yes	yes	no	no	no
16	yes	yes	yes	yes	yes	yes
17	yes	yes	yes	yes	yes	yes
18	yes	no	yes	no	no	no
19	yes	yes	yes	yes	yes	yes
20	yes	yes	yes	yes	yes	yes
21	yes	no	yes	no	no	no
22	yes	yes	yes	yes	yes	yes
23	yes	yes	yes	no	no	yes
24	yes	yes	yes	no	no	yes
25	yes	yes	yes	yes	yes	yes
26	no	yes	no	no	no	no
27	no	no	yes	no	no	no
28	yes	yes	yes	no	no	yes
29	yes	yes	yes	yes	yes	yes
30	no	no	no	no	no	no
31	yes	yes	no	no	no	no
32	no	no	yes	no	no	no
Number of participants for whom this segment was reconstructed	24	25	24	13	16	20

Table 4A. *Results of partial correlational analysis (Pearson's r) between tract metrics of the left arcuate fasciculus (AF) and language indices accounting only for gender.*

Left hemisphere		AF all	AF long	AF anterior	AF posterior	AF-STG	AF-MTG	AF-Temp. Pole
		Normalized volume						
Lesion volume		-.62**	-.60**	-.59**	-.19	-.59**	-.57	-.45*
WAB	Fluency	.46*	.43*	.30	.28	.33	.35	.43*
WAB	Information content	.35	.31	.06	.39*	.19	.18	.36*
WAB	Repetition	.51**	.48**	.17	.45*	.40*	.43*	.37*
WAB	Naming	.51**	.42*	.21	.54**	.35	.37*	.51**
WAB	Comprehension	.49*	.43*	.02	.49**	.21	.24	.29
WAB	AQ	.53**	.48**	.20	.49**	.36*	.38*	.47**
BNT		.54**	.49**	.20	.49**	.49**	.52**	.56**
CYCLE	Simple	.39*	.39	.00	.38	.25	.24	.35
CYCLE	Word Order	.39*	.40	-.01	.40	.27	.33	.26
CYCLE	Complex	.53**	.52**	.16	.43	.24	.32	.35
		HMOA						
Lesion volume		-	-.47**	-.68**	-.14	-.50**	-.54**	-.48**
WAB	Fluency	-	.46**	.60**	.39*	.42*	.41*	.44*
WAB	Information content	-	.46*	.24	.49	.23	.24	.34
WAB	Repetition	-	.62**	.38*	.57**	.46**	.49**	.50**
WAB	Naming	-	.53**	.41*	.61**	.39*	.44*	.54*
WAB	Comprehension	-	.57**	.29	.64**	.28	.28	.39*
WAB	AQ	-	.61**	.46**	.61**	.43*	.45*	.52**
BNT		-	.50**	.43*	.53**	.58**	.58**	.60**
CYCLE	Simple	-	.43*	.24	.50**	.35	.22	.31
CYCLE	Word Order	-	.46*	.25	.46*	.38*	.42*	.39*
CYCLE	Complex	-	.52**	.37*	.60**	.38*	.37*	.39*

Note. * $p < .05$ (in the color version correlations significant at this level are shown in black boldface),
** $p < .01$ (in the color version correlations significant at this level are shown in red boldface).

Table 5A. Results of partial correlational analysis (Pearson's r) between tract metrics of the right arcuate fasciculus (AF) and language indices accounting for gender, time post-onset and lesion volume.

Right hemisphere		AF long	AF anterior	AF posterior	AF-STG	AF-MTG	AF-Temp. Pole
		**Normalized volume**					
WAB	**Fluency**	-.18	**.37***	.01	-.07	**-.37***	.11
	**Information content**	-.16	.06	.12	.09	-.35	-.03
	**Repetition**	.00	.15	.01	.14	-.36	-.03
	**Naming**	-.17	-.01	.16	.09	-.29	.11
	**Comprehension**	-.24	.10	-.04	.03	**-.40***	.16
	**AQ**	-.17	.16	.07	.08	**-.41***	.07
**BNT**		-.09	.17	.23	.23	-.15	.05
CYCLE	**Simple**	-.09	.28	.06	.03	-.35	.09
	**Word Order**	-.26	.01	.16	.23	-.30	-.11
	**Complex**	-.15	.19	.08	.19	-.21	.09
		**HMOA**					
WAB	**Fluency**	-.03	-.03	.05	-.01	-.19	.18
	**Information content**	-.01	-.13	.00	-.02	-.16	-.09
	**Repetition**	.09	-.10	-.10	.19	-.01	.15
	**Naming**	-.01	-.19	-.03	-.06	-.11	.07
	**Comprehension**	.11	.01	-.01	-.07	-.08	.26
	**AQ**	.03	-.12	-.03	.02	-.13	.12
**BNT**		.02	-.09	.08	.15	-.10	.20
CYCLE	**Simple**	.09	.32	.03	-.01	-.10	.25
	**Word Order**	.17	.05	.17	.16	-.07	.26
	**Complex**	.32	.16	.26	.00	.06	**.46***

Note. * $p < .05$, uncorrected (in the color version correlations significant at this level are shown in black boldface),
** $p < .05$, FDR corrected (in the color version correlations significant at this level are shown in red boldface).
